# Supplementary material for: GSK3-CRMP2 signaling mediates axonal regeneration induced by Pten knockout
Source: Commun Biol. 2019 Aug 23;2:318. doi: 10.1038/s42003-019-0524-1 (PMC6707209; doi:10.1038/s42003-019-0524-1)
Supplement: Supplementary file 3 — Reporting Summary [file 42003_2019_524_MOESM3_ESM.pdf]

nature research

Corresponding author(s): Dietmar Fischer

Last updated by author(s): Jun 16, 2019

Reporting Summary

Nature Research wishes to improve the reproducibility of the work that we publish. This form provides structure for consistency and transparency in reporting. For further information on Nature Research policies, see Authors & Referees and the Editorial Policy Checklist.

Statistics

For all statistical analyses, confirm that the following items are present in the figure legend, table legend, main text, or Methods section.

n/a	Confirmed	
☐	☒	The exact sample size (*n*) for each experimental group/condition, given as a discrete number and unit of measurement
☐	☒	A statement on whether measurements were taken from distinct samples or whether the same sample was measured repeatedly
☐	☒	The statistical test(s) used AND whether they are one- or two-sided<br>*Only common tests should be described solely by name; describe more complex techniques in the Methods section.*
☒	☐	A description of all covariates tested
☐	☒	A description of any assumptions or corrections, such as tests of normality and adjustment for multiple comparisons
☐	☒	A full description of the statistical parameters including central tendency (e.g. means) or other basic estimates (e.g. regression coefficient) AND variation (e.g. standard deviation) or associated estimates of uncertainty (e.g. confidence intervals)
☐	☒	For null hypothesis testing, the test statistic (e.g. *F*, *t*, *r*) with confidence intervals, effect sizes, degrees of freedom and *P* value noted<br>*Give P values as exact values whenever suitable.*
☒	☐	For Bayesian analysis, information on the choice of priors and Markov chain Monte Carlo settings
☒	☐	For hierarchical and complex designs, identification of the appropriate level for tests and full reporting of outcomes
☒	☐	Estimates of effect sizes (e.g. Cohen's *d*, Pearson's *r*), indicating how they were calculated

Our web collection on statistics for biologists contains articles on many of the points above.

Software and code

Policy information about availability of computer code

Data collection	Axiovision 4.9 Zeiss; Zen black Zeiss; LAS X Leica
Data analysis	Sigma STAT 3.2 Systat Software; ImageJ (https://imagej.nih.gov/ij/); Adobe Photoshop CS5

For manuscripts utilizing custom algorithms or software that are central to the research but not yet described in published literature, software must be made available to editors/reviewers. We strongly encourage code deposition in a community repository (e.g. GitHub). See the Nature Research guidelines for submitting code & software for further information.

Data

Policy information about availability of data

All manuscripts must include a data availability statement. This statement should provide the following information, where applicable:

- Accession codes, unique identifiers, or web links for publicly available datasets
- A list of figures that have associated raw data
- A description of any restrictions on data availability

Data Availability Statement
All data generated or analysed during this study are included in this published article (and its supplementary information files).

Field-specific reporting

Please select the one below that is the best fit for your research. If you are not sure, read the appropriate sections before making your selection.

☒ Life sciences ☐ Behavioural & social sciences ☐ Ecological, evolutionary & environmental sciences

For a reference copy of the document with all sections, see nature.com/documents/nr-reporting-summary-flat.pdf

Life sciences study design

All studies must disclose on these points even when the disclosure is negative.

Sample size	The sample size was calculated using Sigma STAT3.2 software with a power of 0.8, alpha of 0.05, minimum detectable difference of 0.25 and expected standard deviation of 0.1.
Data exclusions	We did not exclude data. Only animals which developed a lens Injury, or microphthalmia after surgery were not considered for tissue harvest and evaluation.
Replication	Cell culture experiments were repeated several times independently, while each experiments contained 4 individual wells as technical replicate. In vivo regeneration, western blot an immunohistochemistry data were repeated several times with independent samples from individual animals. All data were reproducible.
Randomization	Male and female littermates were randomly and equally allocated to experimental groups.
Blinding	Investigators were blinded during data collection and analysis.

Reporting for specific materials, systems and methods

We require information from authors about some types of materials, experimental systems and methods used in many studies. Here, indicate whether each material, system or method listed is relevant to your study. If you are not sure if a list item applies to your research, read the appropriate section before selecting a response.

Materials & experimental systems

n/a	Involved in the study	
☐	☒	Antibodies
☒	☐	Eukaryotic cell lines
☒	☐	Palaeontology
☐	☒	Animals and other organisms
☒	☐	Human research participants
☒	☐	Clinical data

Methods

n/a	Involved in the study	
☒	☐	ChIP-seq
☒	☐	Flow cytometry
☒	☐	MRI-based neuroimaging

Antibodies

Antibodies used

polyclonal HA antibody (RRID:AB_260070); polyclonal GFP antibody (RRID:AB_10128178); polyclonal pCRMP2 and pS6 antibodies (RRID:AB_942229; RRID:AB_2181035); antibodies against βIII-tubulin (RRID:AB_2313773);S9-phosphorylated GSK3β (RRID:AB_2115196); pS21-GSK3a (RRID:AB_11166651); total GSK3 (RRID:AB_10547140); total CRMP2 (RRID:AB_2094339);T308-phosphorylated AKT (RRID:AB_2629447)

Validation

polyclonal HA antibody (RRID:AB_260070):
Validation:
Validated in western blot and immunohistochemistry according to the manufacturer's website:
"Anti-HA antibody is suitable for use in immunoprecipitation and western blot. (Ludwig M, et al. Human Genetics 117(2-3), 228-237, (2005); Iris H Henn et. al The Journal of neuroscience, 27(8), undefined (2007-2-23))
It is also suitable for indirect immunofluorescence (10-20µg/mL using HA-tagged fusion protein transfected cells), immunoprecipitation (2.5-4µg/test using HA-tagged fusion protein from cell lysates), and western blot (0.5-0.8µg/mL using HA-tagged fusion protein transfected cell extracts)."
Target:
HA human
Publications:
PMID:21830221, PMID:27538435, PMID:27637097, PMID:27725085, PMID:28630333, PMID:28841137, PMID:28951451, PMID:29024645, PMID:29024665, PMID:29073373, PMID:29154130, PMID:29290488, PMID:29290611, PMID:29649442, PMID:29677490, PMID:29681460, PMID:29934346, PMID:29983322, PMID:30044983, PMID:30078703, PMID:30231999, PMID:30244971, PMID:30281024, PMID:30293784, PMID:30404010, PMID:30404013, PMID:30415698, PMID:30550789, PMID:30612859, PMID:30784600, PMID:30995488

polyclonal GFP antibody (RRID:AB_10128178):
Validation:
Seller suggested use: ELISA; Immunohistochemistry; Immunohistochemistry - fixed; Immunofluorescence; Western Blot; ELISA, Immunofluorescence, Immunohistochemistry, Immunohistochemistry-Paraffin, Western Blot.
Publications:
PMID:28600222, PMID:28630333, PMID:28641114, PMID:29222469, PMID:29587142, PMID:30454645, PMID:30982771

polyclonal pCRMP2 antibody (RRID:AB_942229):
Validation:
Seller recommendations provided in 2012: ELISA; Immunocytochemistry; Immunofluorescence; Immunohistochemistry; ELISA, Immunocytochemistry/Immunofluorescence, Immunohistochemistry-P, Western Blot.
Target:
CRMP2 (phospho T514)human, mouse, rat
Publications:
PMID:28630333

monoclonal pS6 antibody (RRID:AB_2181035):
Validation:
Validated according to manufacturer's homepage by "western blot analysis of extracts from NIH/3T3 cells, untreated or PDGF-treated (100ng/ml, 20 min)".
Seller suggested use: Western Blot, Immunofluorescence, Immunohistochemistry, Immunohistochemistry-Paraffin.
Target:
Phospho-S6 Ribosomal Protein (Ser235/236) See NCBI gene human, mouse, rat
Publications:
PMID:25100599, PMID:27641504, PMID:28641110, PMID:28817801, PMID:28880149, PMID:29695414, PMID:29727620, PMID:30673598

monoclonal βIII-tubulin antibody (RRID:AB_2313773):
Validation:
According to manufacturer's homepage "This antibody is well characterized and highly reactive to neuron specific Class III β-tubulin (βIII). TUJ1 does not identify β-tubulin found in glial cells. TUJ1 recognizes an epitope located within the last 15 C-terminal residues.
Nishimura K, et al. 2017. PLoS One. 12(1): e0170568. (ICC)
Jongbloets J, et al. 2017. Nat Commun. 8: 14666. (ICC) PubMed
Liu W.J, et al. 2015. Eur J Histochem. 59(1): 2464. (ICC) PubMed
Chintalapudi SR, et al. 2016. Front Aging Neursci. 8:93. (FC, ICC) PubMed
Ambasudhan R, et al. 2011. Cell Stem Cell. 9(2):113. (IHC, ICC)
Hu X., et al. 2006. Nature Neuroscicene. 9(12):1520. (WB) PubMed
Zechner D., et al. 2003. Develop Biology. 258(2):406. (ICC, IHC)
Lee MK, et al. 1990. Proc. Natl. Acad. Sci. USA 18:7195. (WB)"
Target:
Neuronal Class III beta-Tubulin (TUJ1) Purified mammalian, other mammalian, hamster, sheep, bovine, horse, rabbit, guinea pig, human, non-human primate, donkey, feline, goat, porcine, canine, mouse, rat
Publications:
PMID:16680766, PMID:16786555, PMID:16977618, PMID:17154269, PMID:17335037, PMID:17436285, PMID:18205207, PMID:18271024, PMID:18551532, PMID:18651636, PMID:18729150, PMID:18803239, PMID:19350672, PMID:19399895, PMID:19479999, PMID:20017208, PMID:20506477, PMID:21344404, PMID:21452215, PMID:22522921, PMID:22806400, PMID:22847514, PMID:23640803, PMID:25795781, PMID:25961839, PMID:26898779, PMID:27018986, PMID:27418162, PMID:27644593, PMID:27779093, PMID:28009275, PMID:28079521, PMID:28111074, PMID:28123024, PMID:28132826, PMID:28238547, PMID:28340341, PMID:28377582, PMID:28380383, PMID:28426964, PMID:28440222, PMID:28457792, PMID:28483977, PMID:28512649, PMID:28535372, PMID:28539419, PMID:28543060, PMID:28552557, PMID:28630333, PMID:28641113, PMID:28669631, PMID:28789474, PMID:28817799, PMID:28943241, PMID:28965825, PMID:29024661, PMID:29034884, PMID:29151587, PMID:29168882, PMID:29207259, PMID:29218724, PMID:29225067, PMID:29249360, PMID:29249622, PMID:29290548, PMID:29503187, PMID:29551301, PMID:29631040, PMID:29656178, PMID:29660608, PMID:29677589, PMID:29684900, PMID:29706593, PMID:29712777, PMID:29749639, PMID:29784083, PMID:29788427, PMID:29807259, PMID:29853629, PMID:29854941, PMID:29887339, PMID:29906669, PMID:29911975, PMID:29934351, PMID:29961574, PMID:29974865, PMID:30017396, PMID:30057116, PMID:30078710, PMID:30086304, PMID:30099334, PMID:30134160, PMID:30184491, PMID:30225353, PMID:30232223, PMID:30246867, PMID:30252950, PMID:30308165, PMID:30318302, PMID:30343101, PMID:30344048, PMID:30415925, PMID:30449657, PMID:30454561, PMID:30483071, PMID:30485816, PMID:30503143, PMID:30562514, PMID:30562574, PMID:30625321, PMID:30644360, PMID:30650353, PMID:30661738, PMID:30673598, PMID:30697732, PMID:30699345, PMID:30699346, PMID:30735633, PMID:30739799, PMID:30770246, PMID:30846309, PMID:30878013, PMID:30878014, PMID:30891830, PMID:30893594, PMID:30905607, PMID:30921587, PMID:30943410, PMID:31030416, PMID:31042147, PMID:31067457, PMID:31091456, PMID:31099332, PMID:31124784

polyclonal S9-phosphorylated GSK3β antibody (RRID:AB_2115196):
Validation:
Validated in western blot and immunohistochemistry by absence of signal in GSK3αS21/A/βS9/A mice with non-phosphorylatable GSK3 in this manuscript and in previous publications (Leibinger et al., 2017).
Target:
Gsk3b human, mouse, rat
Publikations:
PMID:28630333, PMID:29547721

pS21-GSK3α (RRID: AB_11166651):

Validation:
Validated in western blot and immunohistochemistry by absence of signal in GSK3αS21/A/βS9/A mice non- phosphorylatable GSK3 in this manuscript and in previous publications (Leibinger et al., 2017).
Manufacturer recommendations: Immunocytochemistry; Immunofluorescence; Immunohistochemistry; Western Blot.
Target:
GSK3 alpha (Phospho Ser21) mouse, human, rat, human, mouse, rat
Gsk3a human, rat, mouse
Publications:
PMID:28630333

total GSK3 (RRID: AB_10547140):
Validation:
Validated according to manufacturer's homepage by "Western blot analysis of extracts from HeLa cells, mock transfected or transfected with SignalSilence® GSK-3α/β siRNA #6301". Applications: W, IP. Consolidation on 9/2016: AB_10859043.
Target:
GSK-3alpha/beta (D75D3) XP Rabbit mAb non-human primate, mouse, rat, hamster, human, h, m, r, hm, mk
Publications:
PMID:26280128, PMID:28374012, PMID:28757207, PMID:29149593, PMID:29763624, PMID:29861159, PMID:29910151, PMID:30471094, PMID:30704899, PMID:30825187, PMID:30991026

total CRMP2 (RRID:AB_2094339):
Validation:
Seller suggested use: Immunohistochemistry; Immunofluorescence; Western Blot
Target:
CRMP-2 human, mouse, rat
Publications:
PMID:28630333, PMID:30562574

T308-phosphorylated AKT (RRID:AB_2629447):
Validation:
According to manufacturer's homepage, this antibody was validated in "Western blot analysis of extracts from NIH/3T3 cells, untreated (-) or treated with Human Platelet-Derived Growth Factor AA (hPDGF-AA) #8913 (100 ng/ml, 5 min; +), and untreated (-) LNCaP and PC-3 cells. Phospho-Akt (Thr308) (D25E6) XP® Rabbit mAb recognizes endogenous levels of Akt1 protein only when phosphorylated at Thr308. This antibody also recognizes endogenous levels of Akt2 protein when phosphorylated at Thr309 or Akt3 protein when phosphorylated at Thr305. "
Target:
Phospho-Akt (Thr308) Human, Mouse, Rat, Monkey
Publications:
PMID:27792406, PMID:28406396, PMID:28630333, PMID:28757207, PMID:28919041, PMID:29029116, PMID:29246441, PMID:30029001, PMID:30078705, PMID:30126838, PMID:30174303, PMID:30300582, PMID:30566860, PMID:30581121, PMID:30639242, PMID:30704899, PMID:30734931, PMID:30738829, PMID:30773462, PMID:30784600, PMID:31091439

Animals and other organisms

Policy information about studies involving animals; ARRIVE guidelines recommended for reporting animal research

Laboratory animals	Male and female mice (2-3 months old) were used. Genotypes: GSK3b Ser9/Ala; GSK3a Ser21/Ala; GSK3b Ser9/Ala /a Ser21/Ala; all C57BL/6,129/Ola background and respective wt C57BL/6,129/Ola mice. These mice were also crossbred with PTENf/f mice (C57BL/6;129) mice. Moreover, GSK3βf/f , GSK3αf/f and GSK3α/βf/f mice (C57BL/6;129) were used and also crossbred with PTENf/f mice.
Wild animals	not used
Field-collected samples	not used
Ethics oversight	All experimental procedures were approved by the local animal care committee (LANUV Recklinghausen) and conducted in compliance with federal and state guidelines for animal experiments in Germany.

Note that full information on the approval of the study protocol must also be provided in the manuscript.
