# Supplementary material for: GSK3-CRMP2 signaling mediates axonal regeneration induced by Pten knockout
Source: Commun Biol. 2019 Aug 23;2:318. doi: 10.1038/s42003-019-0524-1 (PMC6707209; doi:10.1038/s42003-019-0524-1)
Supplement: Supplementary file 1 — Supplementary Information [file 42003_2019_524_MOESM1_ESM.pdf]

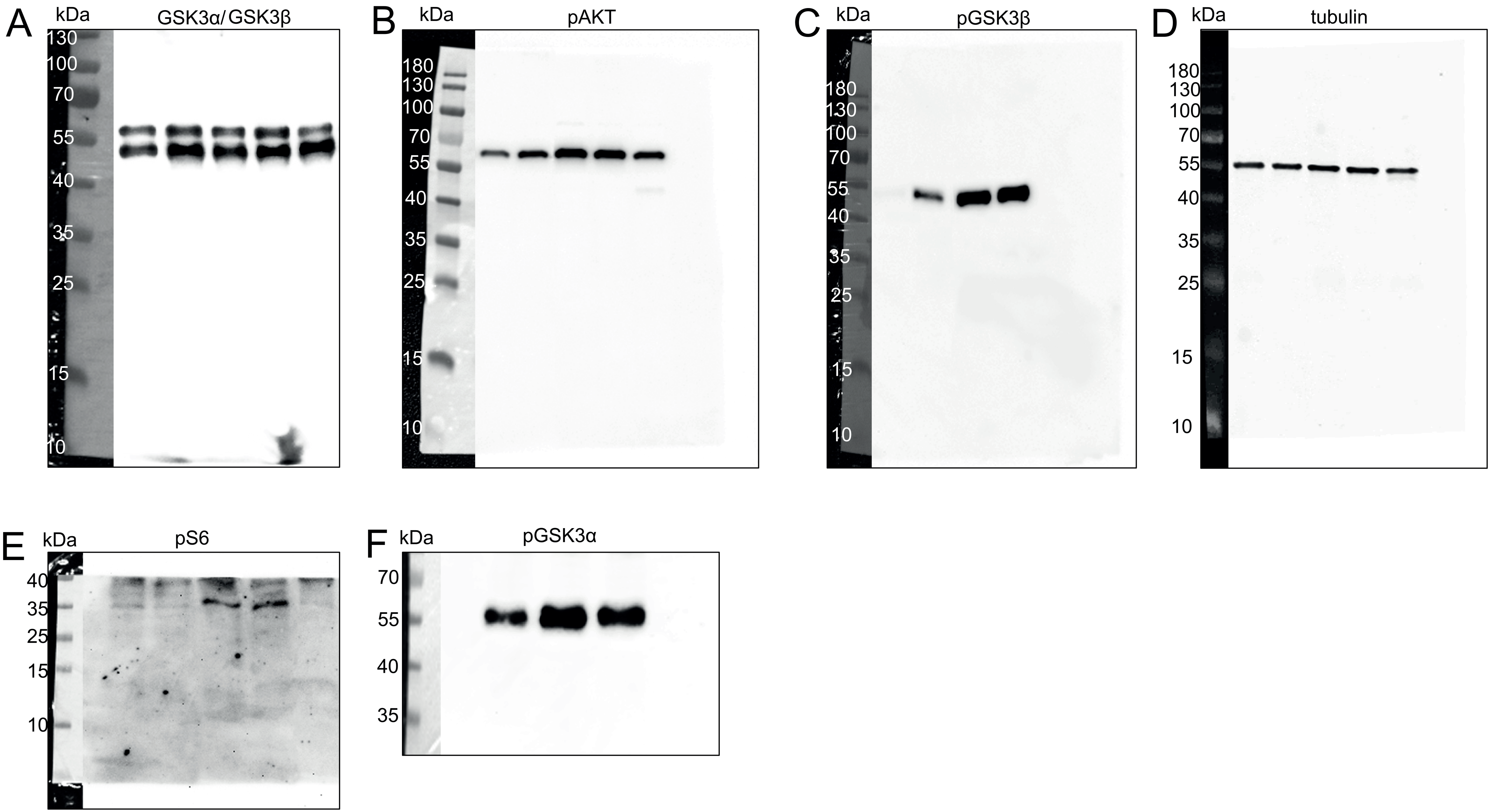

Supplementary Figure 1: Membranes from western blots shown in Fig. 1D

Original Western Blot images including protein ladder from blots for total GSK3α (upper band) and β (lower band) (A), phosphorylated AKT (B), phospho-rylated GSK3β (C), βIII-tubulin (D), phosphorylated S6 (E) and phosphorylated GSK3α (F) as shown in Fig. 1 D.

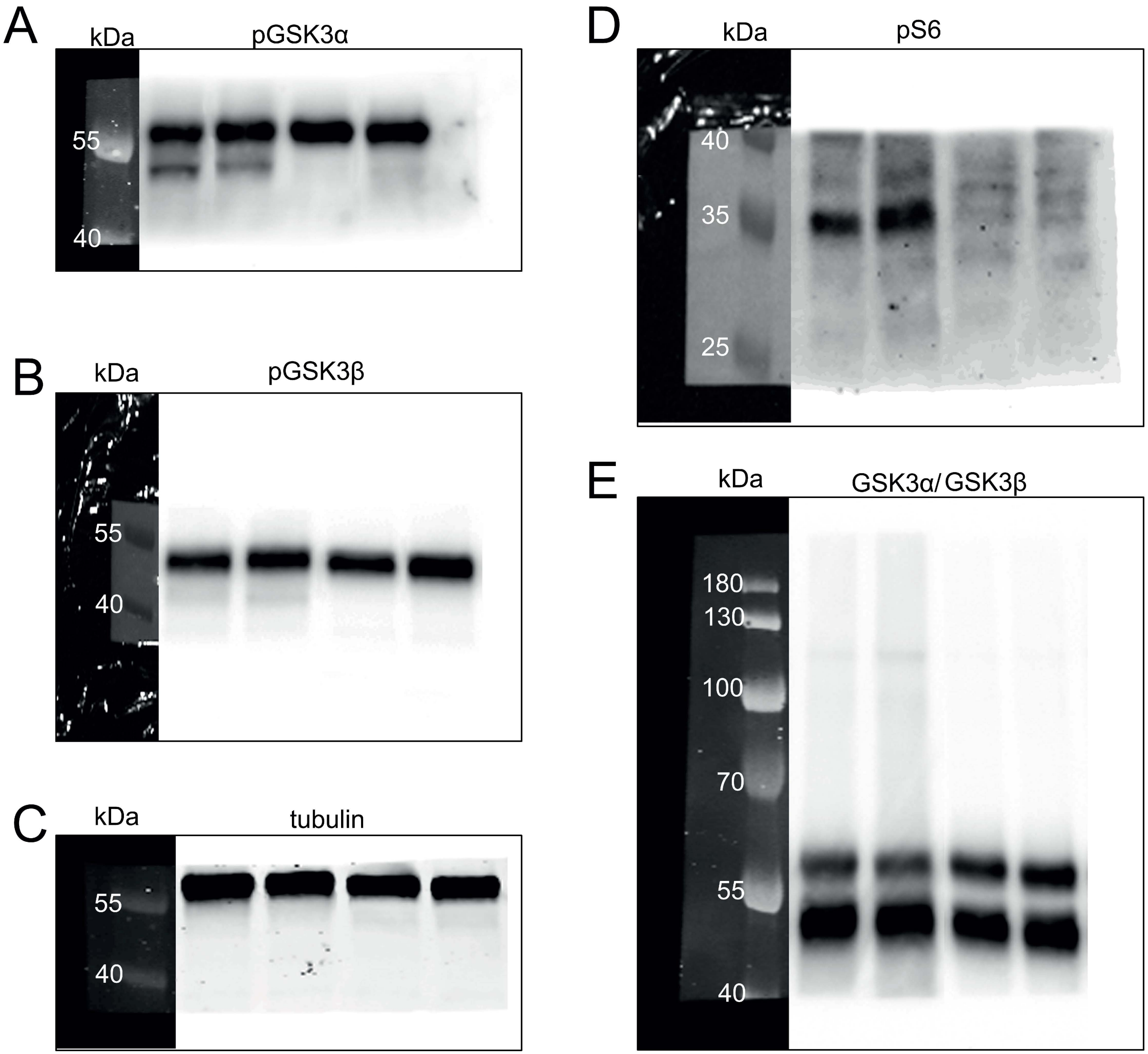

Supplementary Figure 2: Membranes from western blots shown in Fig. 2 b
Original images from western blot membranes including protein ladder from blots for phosphorylated GSK3 α (A), phosphorylated GSK3β (B), βIII-tubulin (C), phosphorylated S6 (D) and total GSK3α (upper band) and β (lower band) (E).

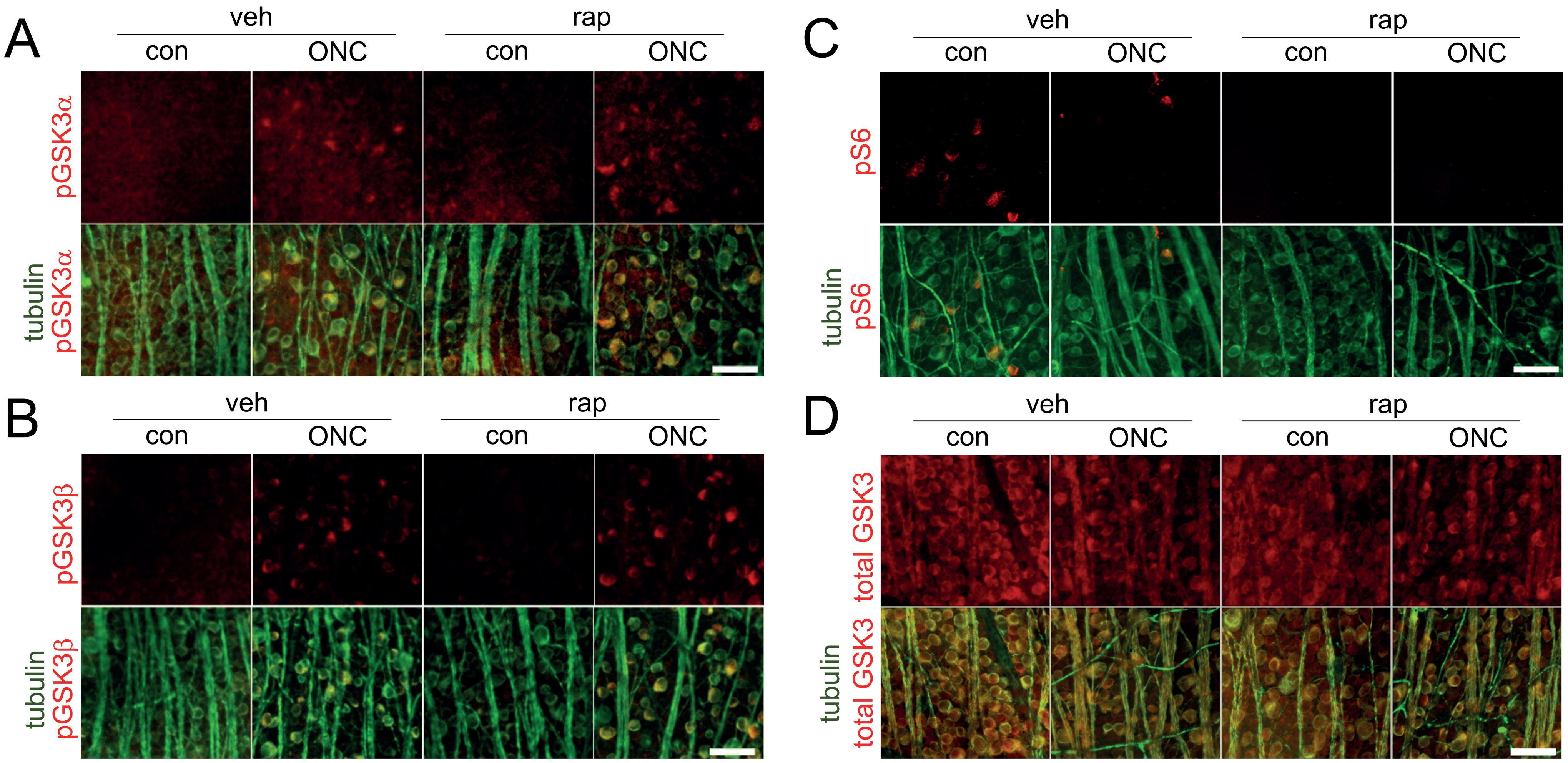

Supplementary Figure 3: ONC induced GSK3 phosphorylation is mTOR independent
A-D) Retinal wholemounts of wild type mice after intraperitoneal injection of either vehicle (veh) or rapamycin (rap). Animals remained either untreated (con) or received ONC 5 days before tissue isolation. ONC induced S21-phosphorylation of GSK3α (pGSK3α, red, A) and S9-phosphorylation of GSK3β (pGSK3β, red, B) in βIII-tubulin-positive RGCs (green) independently of rapamycin treatment, which abolished S6 phosphorylation (pS6, red, C). Total GSK3 protein expression (total GSK3, red, D) remained unaltered among all treatment groups. Stainings represented in A-D were repeated with 4 independent retinae per group showing the same results. Scale bars: 50 µm.

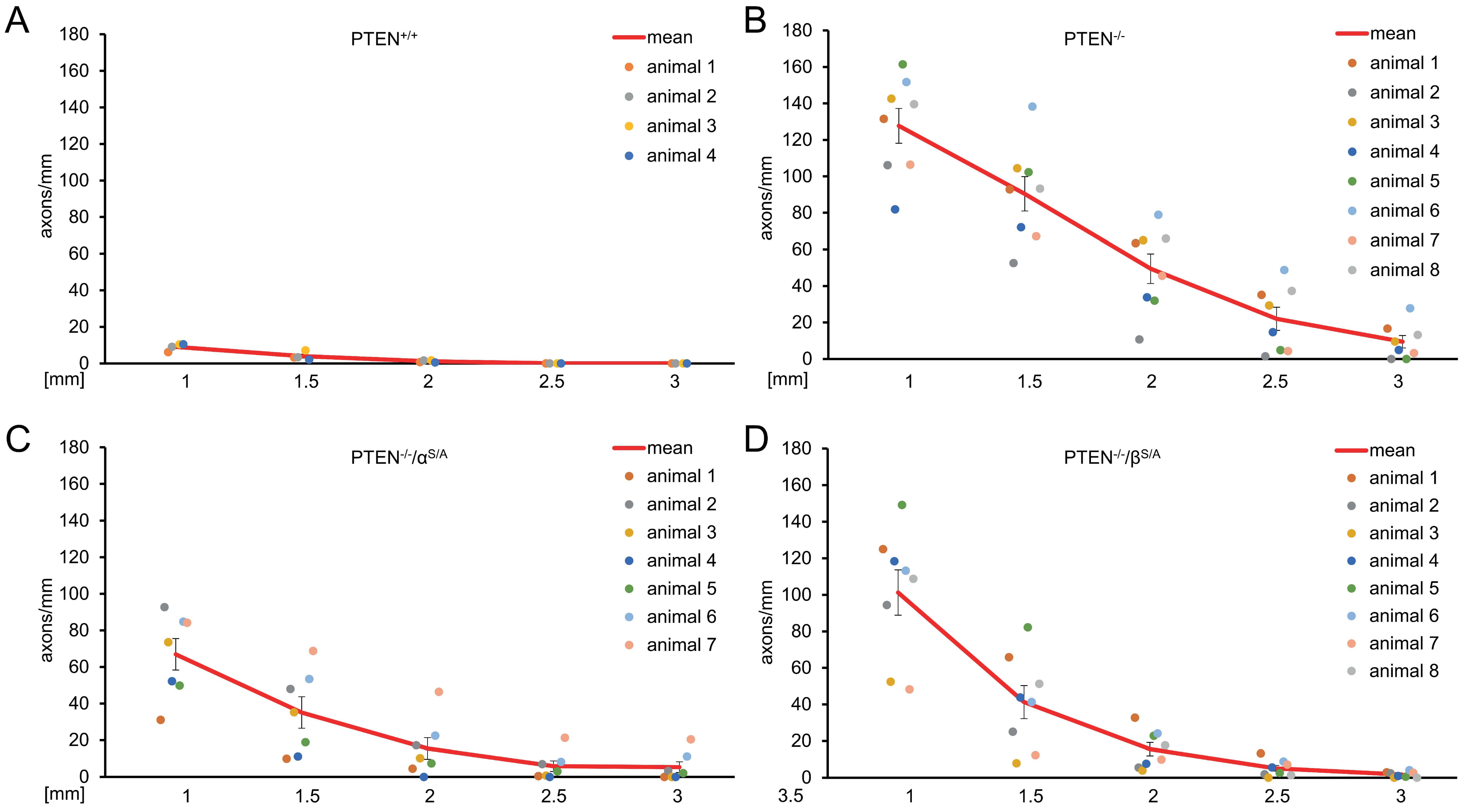

Supplementary Figure 4: Single values of data presented in Fig. 3 H
Values of regenerating axons per millimeter of optic nerve at 1, 1.5, 2, 2.5, and 3 mm beyond the lesion site used for the in vivo optic nerve regeneration study presented in Fig. 3H in relation to the respective mean value (mean) from the following groups: $PTEN^{+/+}$ (A), $PTEN^{-/-}$, (B) $PTEN^{-/-}/\alpha^{S/A}$ (C), PTEN-/-/$\beta^{S/A}$ (D).

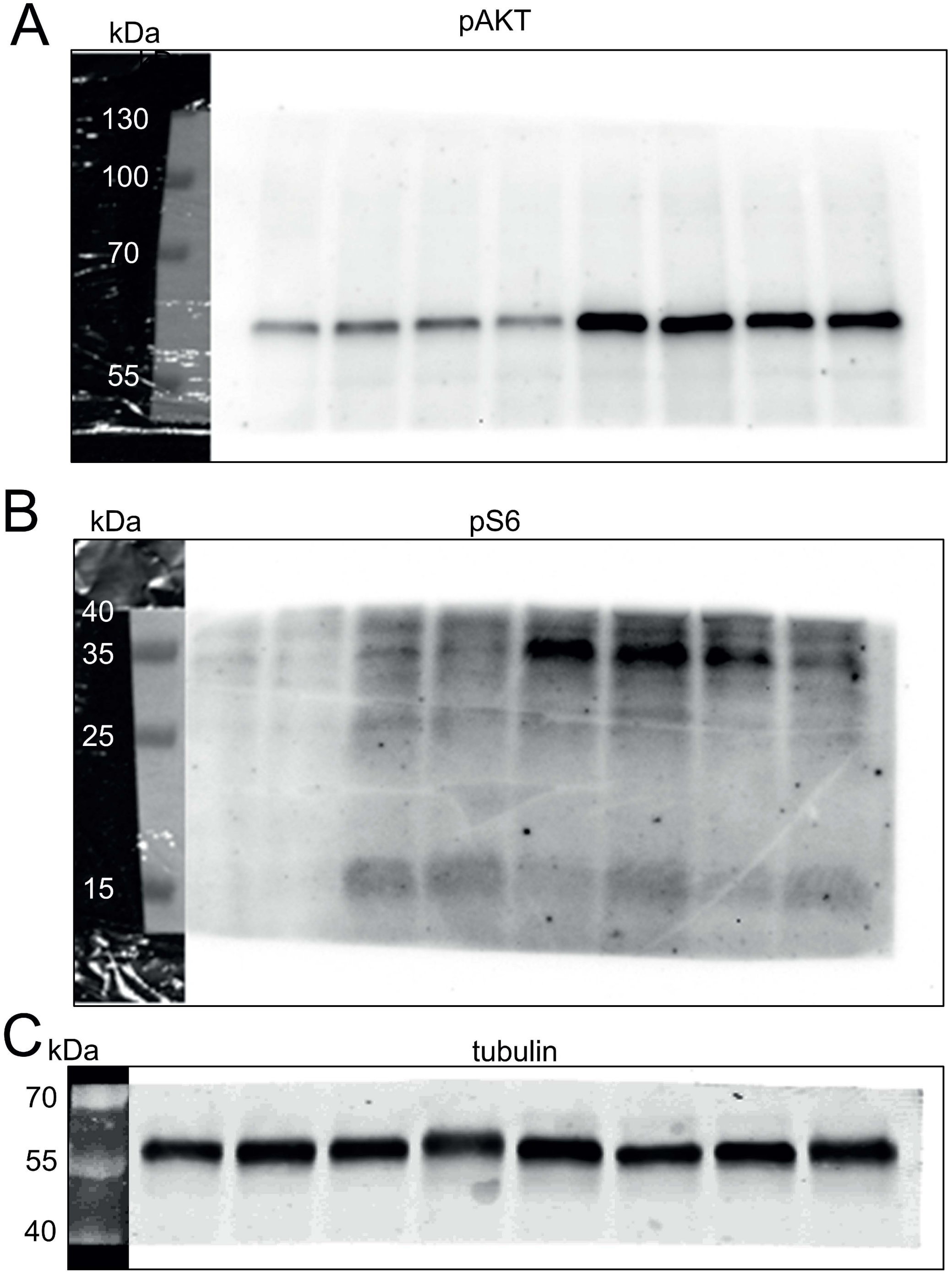

Supplementary Figure 5: Membranes from western blots shown in Fig. 5 D
Original images from western blot membranes including protein ladder from blots for phosphorylated AKT (A), phosphorylated S6 (B) and βIII-tubulin (C) as presented in Fig 5 D.

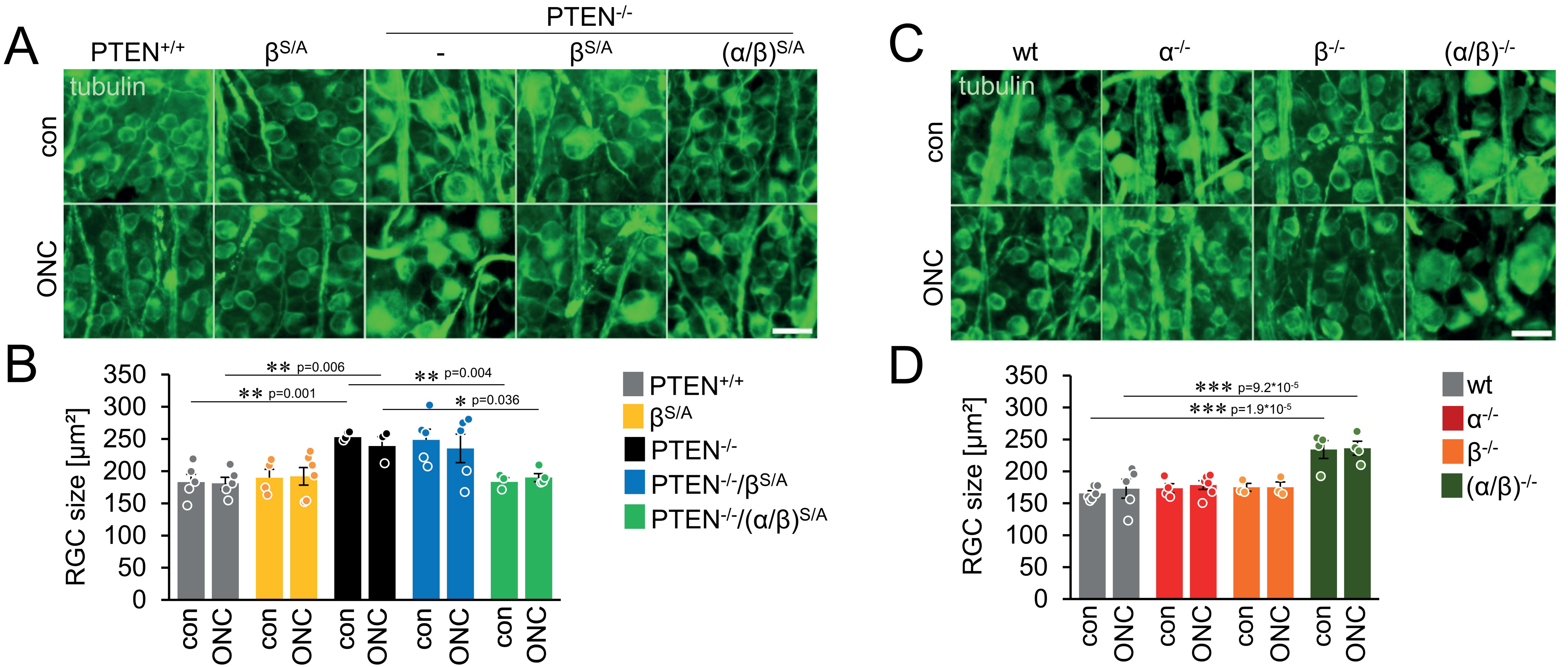

Supplementary Figure 6: PTEN$^{-/-}$ mediated increase in RGC size is GSK3 dependent

A) Retinal flat mounts isolated from PTEN$^{+/+}$/GSK3(α/β)$^{+/+}$ (PTEN$^{+/+}$), GSK3β$^{S/A}$ (β$^{S/A}$), PTEN$^{-/-}$, PTEN$^{-/-}$/GSK3β$^{S/A}$ and PTEN$^{-/-}$/GSK3(α/β)$^{S/A}$ mice which were either left untreated (con) or subjected to ONC. Five days after surgery, retinae were fixed and stained for βIII-tubulin (tubulin; green) to visualize RGCs. Scale bar: 25 μm.
B) Quantification of average soma sizes from RGCs in flat mounts as depicted in A. Compared to wt animals, average sizes were markedly increased after PTEN$^{-/-}$ and significantly compromised in animals with additional GSK3(α/β)$^{S/A}$ knockin, but not GSK3β$^{S/A}$ single knockin. Values represent means +/- SEM of 3-6 retinae with ~200 RGCs per group (untreated controls: PTEN$^{+/+}$, n=5; β$^{S/A}$, n=4; PTEN$^{-/-}$, n=4; PTEN$^{-/-}$/βS/A, n=5; PTEN$^{-/-}$/(α/β)$^{S/A}$, n=3; mice 5 d after ONC: PTEN$^{+/+}$, n=5; β$^{S/A}$, n=6; PTEN$^{-/-}$, n=3; PTEN$^{-/-}$/β$^{S/A}$, n=5; PTEN$^{-/-}$/(α/β)$^{S/A}$, n=4).
C) Retinal flat mounts isolated from GSK3(α/β)$^{+/+}$ (wt), GSK3α$^{-/-}$ (α$^{-/-}$), GSK3β$^{-/-}$ (β$^{-/-}$), GSK3(α/β)$^{-/-}$ ((α/β)$^{-/-}$), mice which were either left untreated (con) or subjected to ONC. Five days after surgery, retinae were fixed and stained for βIII-tubulin (tubulin; green) to visualize RGCs. Scale bar: 25 μm.
D) Quantification of soma size from RGCs in flat mounts as depicted in C. Compared to wt animals, RGC size was markedly increased after GSK3(α/β)$^{-/-}$, but not GSK3α$^{-/-}$ or GSK3β$^{-/-}$ single knockouts. Values represent means +/- SEM of 3-6 retinae with ~200 RGCs per group (untreated controls: wt n=6; β$^{-/-}$, n=3; α$^{-/-}$, n= 4; (α/β)$^{-/-}$, n=4; mice 5 d after ONC: wt, n=5; β$^{-/-}$, n=3; α$^{-/-}$, n= 6; (α/β)$^{-/-}$, n=4).
Significances of intergroup differences in B and D were evaluated using one-way analysis of variance (ANOVA) followed by Tukey post hoc test. Treatment effects: *$p<0.05$, **$p<0.01$, ***$p<0.001$.

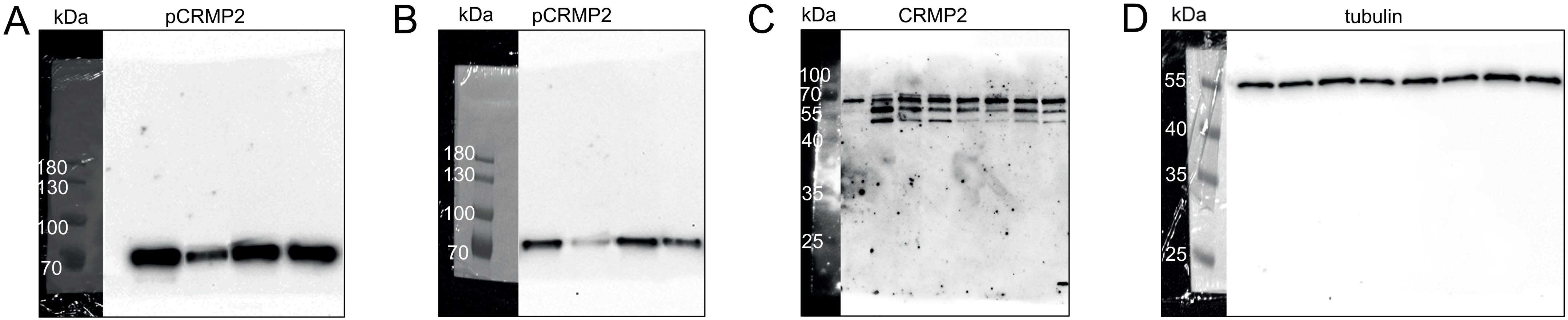

Supplementary Figure 7: Membranes from western blots shown in Fig. 8
Original images from western blot membranes including protein ladder from blots for phosphorylated (A, B) and total CRMP2 (C) and βIII-tubulin (D) as presented in Fig. 8 B and E.

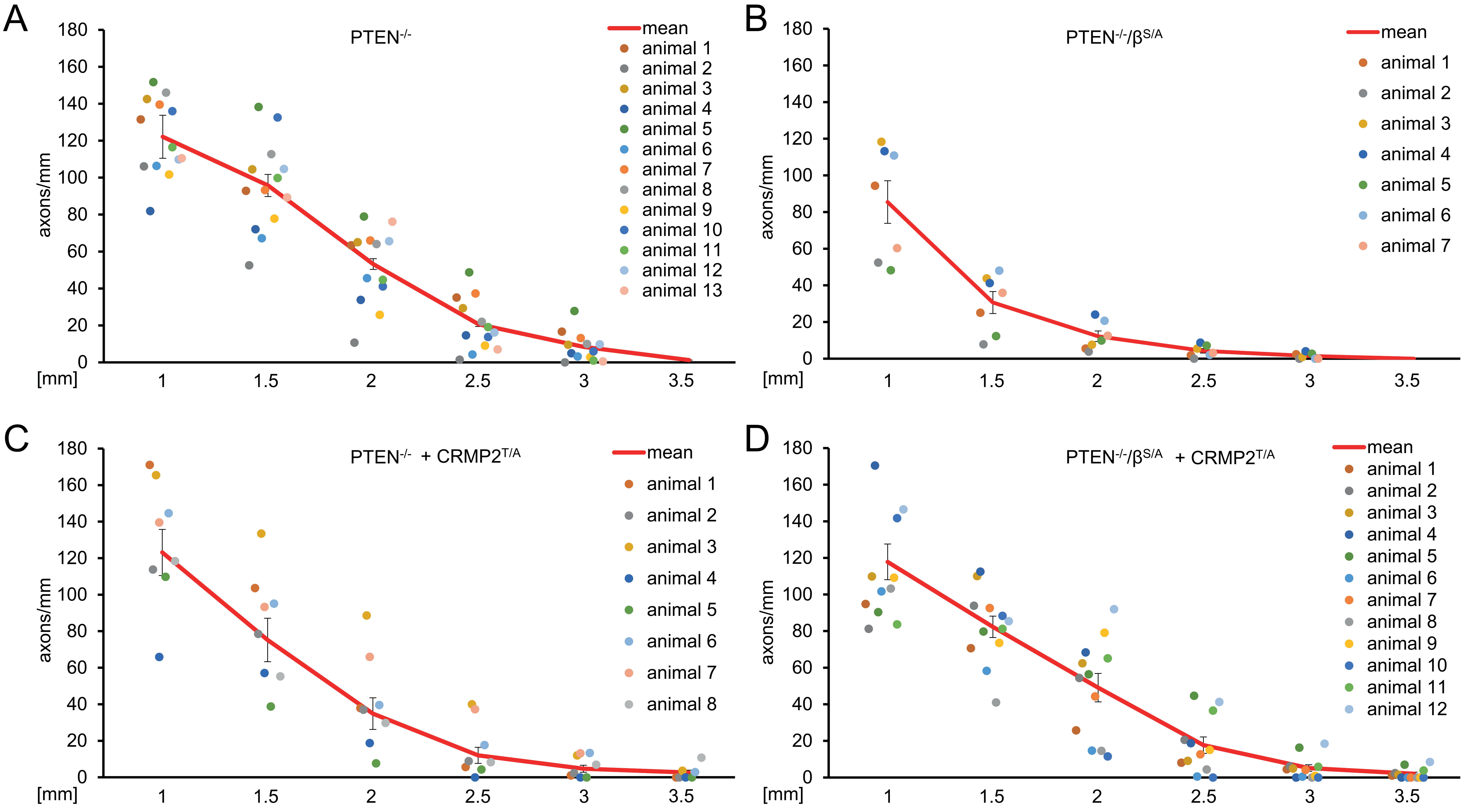

Supplementary Figure 8: Single values of data presented in Fig. 10 G

Values of regenerating axons per millimeter of optic nerve at 1, 1.5, 2, 2.5, and 3 mm beyond the lesion site used for the in vivo optic nerve regeneration study presented in Fig. 10 G in relation to the respective mean value (mean) from the following groups: PTEN$^{-/-}$ (A), PTEN$^{-/-}$/β$^{S/A}$ (B), PTEN$^{-/-}$ + CRMP2$^{T/A}$, (C), PTEN$^{-/-}$/β$^{S/A}$ + CRMP2$^{T/A}$ (D).
